# Clinical yield of serial follow-up by stress CMR in high cardiovascular risk patients

**DOI:** 10.3389/fcvm.2022.995752

**Published:** 2022-08-29

**Authors:** Théo Pezel, Philippe Garot, Thierry Unterseeh, Thomas Hovasse, Francesca Sanguineti, Solenn Toupin, Stéphane Morisset, Stéphane Champagne, Jérôme Garot

**Affiliations:** ^1^Institut Cardiovasculaire Paris Sud, Cardiovascular Magnetic Resonance Laboratory, Hôpital Privé Jacques CARTIER, Ramsay Santé, Massy, France; ^2^Université de Paris Cité, Service de Cardiologie, Hôpital Lariboisière—APHP, Inserm UMRS 942, Paris, France; ^3^Siemens Healthcare France Saint-Denis, Bavaria, France; ^4^Independent Biostatistician, Pérouges, France

**Keywords:** cardiovascular magnetic resonance (CMR), stress test, follow-up, prognosis, death

Stress cardiovascular magnetic resonance (CMR) is accurate and cost-effective for risk stratification, notably through the detection of inducible ischemia and myocardial scar (1, 2). In patients with suspected coronary artery disease (CAD), studies have shown the excellent negative predictive value of a normal stress CMR (defined by the absence of ischemia or myocardial scar) with a 3-year annual event rate <1% (1). Recent studies emphasized the prognostic value of stress CMR in patients without known CAD (3). Although, current guidelines state that it is possible to propose a non-invasive stress test to detect silent ischemia in an asymptomatic patient every 3 to 5 years (4), no study has formally evaluated serial stress CMR assessment in asymptomatic patients. This study aimed to assess the prognostic yield of serial follow-up by stress CMR in asymptomatic high-risk patients.

From December 2008-January 2018, we conducted a longitudinal study with retrospective enrolment of all consecutive asymptomatic patients with ≥2 cardiovascular risk factors (including diabetes, hypertension, current smoking, dyslipidemia, and family history of CAD), but without known CAD, who underwent a normal index stress CMR (1.5T) from our single center with a high volume of stress CMR exams (>3,700/year). Normal stress CMR was defined by the absence of ischemia or late-gadolinium enhancement (LGE). Hyperemia was induced with dipyridamole (0.84 mg/kg over 3 min) after cine imaging (3, 5). A bolus of 0.1 mmol/kg gadolinium chelates was injected (5 ml/s) with acquisitions of 4 left ventricular short-axis and 2 long-axis views using first-pass perfusion imaging. Cross-registered LGE images were acquired 10 min after injection. The analysis of perfusion images was done visually by 2 blinded experienced operators. The definition of ischemia was based on established criteria (5). The follow-up consisted of yearly clinical visits and additional contacts in case of events. The primary outcome was all-cause death using the electronic French National Registry of Death in May 2022. Patients who underwent coronary revascularization ≤ 90 days after CMR exam were censored. The independent association between a second stress CMR in patients still asymptomatic, analyzed as a time-dependent covariate, and the occurrence of all-cause death, was determined by: i) Cox proportional hazards methods with covariables based on clinical input: age, male, obesity, hypertension, diabetes, dyslipidemia, current smoking, renal failure, left ventricular ejection fraction (LVEF), presence of ischemia or LGE on the second CMR, and coronary revascularization after the second CMR; ii) multivariable Cox analysis with adjustment for the propensity score in the overall population; and iii) a separate multivariable Fine and Gray regression analysis (to address the competitive risk analysis between a second CMR study and death) using a 1:1 propensity score-matched population (*n* = 3,078 with only one CMR vs. *n* = 3,078 with 2 serial CMR); and iv) the propensity score method using a doubly robust estimator with augmented inverse propensity score weighting.

Among the 9,377 asymptomatic patients but without known CAD referred for stress CMR (66% men, age 63 ± 12 years), 7,689 (82.0%) patients had a normal index CMR. Among those, 6,996 (91.0%) completed clinical follow-up and 3,086 were still asymptomatic and referred for a second stress CMR (44%, mean 3.7 ± 1.2 years after index CMR), whereas 3,910 were not (56%). The decision to perform a second CMR in these asymptomatic patients was left to the discretion of the referring cardiologist. Regarding cardiovascular risk factors, 54% of patients had hypertension, 52% dyslipidemia, 35% diabetes mellitus, 31% were current or previous smokers, and 11% had a family history of CAD. Overall, 578 (8.2%) patients died at median (IQR) follow-up of 6.8 (5.1–8.8) years. The annual mortality rate was higher in patients who underwent only the index CMR (1.6%/year) vs. patients who had a serial study (0.6%/year, *p* < 0.001). After adjustment, a serial study was independently associated with a lower rate of death (adjusted HR: 0.31; 95% CI: 0.24–0.42, *p* < 0.001, Figure 1A). The variables used for propensity score matching were: age, sex, diabetes, hypertension, dyslipidemia, current smoking, family history of CAD, body mass index, known chronic kidney disease and LVEF value. There were no significant differences in baseline characteristics between the 2 groups matched on propensity score. In the propensity-matched populations, the performance of a second CMR study was associated with a lower death rate (HR: 0.35; 95% CI: 0.26–0.48, *p* < 0.001, Figure 1B). Consistently, after adjustment for the propensity score, a second CMR study was also associated with a lower death-rate (HR: 0.38; 95% CI: 0.27–0.53, *p* < 0.001). Finally, using the propensity score with the doubly robust method, a second CMR study was independently associated a lower death-rate (HR: 0.36; 95% CI: 0.29–0.43, *p* < 0.001).

**Figure 1:**
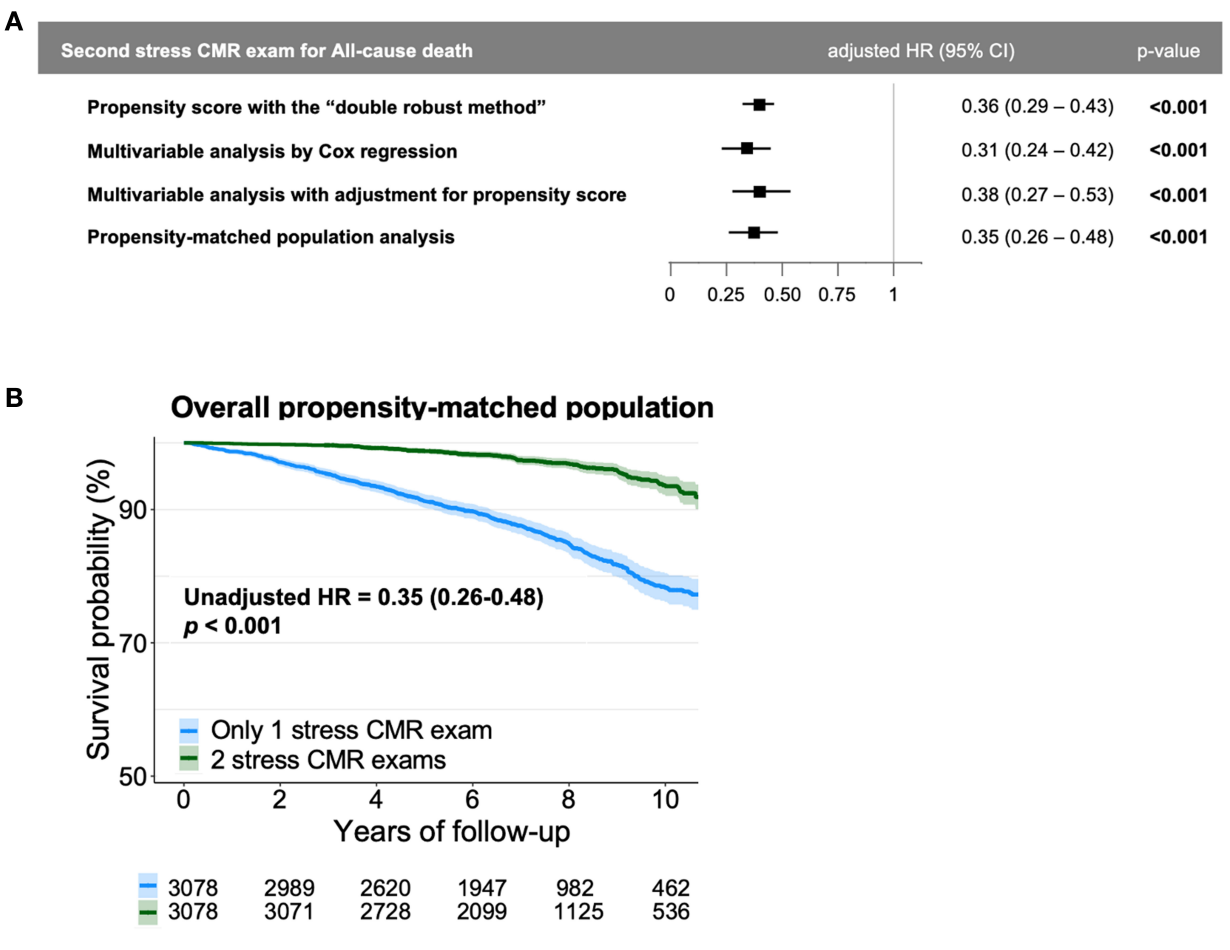
Impact of a second stress CMR study on mortality. **(A)** Forest plots with adjusted hazard ratio for the occurrence of death according to presence/absence of a second stress CMR study. **(B)** Kaplan-Meier curves of all-cause death as a function of follow-up stratified by presence/absence of a second stress CMR study in the propensity-matched population.

Even if no study has formally evaluated serial stress CMR assessment in asymptomatic patients, the current study was in line with prior reports underlining the important negative predictive value of a normal stress CMR (1–3, 5). Regarding study limitations, this was a single-center retrospective study but with a high volume of stress CMR studies (>3,700/year). Baseline data for medications and reasons for the absence of PCI in patients with ischemia were not collected. In addition, the details involved in the decision to perform a second CMR in these asymptomatic patients were not collected. While we had information about the absence of symptoms at the time of the first and second CMR exams, the absence of symptoms was not assessed during the follow-up between the two CMR examinations. However, these limitations were related to patient care and reflect current clinical practice. Although the assessment of all-cause death from the national mortality registry is a robust outcome, it lacks specificity compared to other clinical outcomes such as the assessment of major adverse cardiovascular events. Further randomized clinical trial are required to assess the prognostic impact of serial follow-up stress-CMR studies in high-risk asymptomatic patients, including a cost-effective analysis.

In conclusions, this study shows a potential clinical yield of serial follow-up by stress CMR every 3–5 years in asymptomatic patients at high cardiovascular risk with a first normal index CMR and without known CAD.

## Author contributions

TP and JG conceived the study design. TP, FS, TH, SC, TU, PG, and JG obtained CMR images and analyzed CMR scans. SM performed statistical analyses. TP and JG analyzed data and drafted the manuscript with critical revision. JG and ST have technically defined the CMR protocol and reviewed the technical part of the manuscript. All authors participated in the discussion of the concept of the study. As authors, we attest to each of our substantial contributions to the manuscript and revision. All authors read and approved the final manuscript.

## Conflict of interest

Author ST was employed by Siemens Healthcare. The remaining authors declare that the research was conducted in the absence of any commercial or financial relationships that could be construed as a potential conflict of interest.

## Publisher's note

All claims expressed in this article are solely those of the authors and do not necessarily represent those of their affiliated organizations, or those of the publisher, the editors and the reviewers. Any product that may be evaluated in this article, or claim that may be made by its manufacturer, is not guaranteed or endorsed by the publisher.
